# Epigenetic signature of birth weight discordance in adult twins

**DOI:** 10.1186/1471-2164-15-1062

**Published:** 2014-12-04

**Authors:** Qihua Tan, Morten Frost, Bastiaan T Heijmans, Jacob von Bornemann Hjelmborg, Elmar W Tobi, Kaare Christensen, Lene Christiansen

**Affiliations:** Epidemiology, Biostatistics and Biodemography, Institute of Public Health, University of Southern Denmark, J. B. Winsløws Vej 9B, DK-5000 Odense C, Denmark; Department of Clinical Genetics, Odense University Hospital, Odense, Denmark; Department of Endocrinology, Odense University Hospital, Odense, Denmark; Departments of Molecular Epidemiology and Medical Statistics, Leiden University Medical Center, Odense, Leiden, The Netherlands

**Keywords:** Identical twins, Birth weight discordance, DNA methylation

## Abstract

**Background:**

A low birth weight has been extensively related to poor adult health outcomes. Birth weight can be seen as a proxy for environmental conditions during prenatal development. Identical twin pairs discordant for birth weight provide an extraordinary model for investigating the association between birth weight and adult life health while controlling for not only genetics but also postnatal rearing environment. We performed an epigenome-wide profiling on blood samples from 150 pairs of adult monozygotic twins discordant for birth weight to look for molecular evidence of epigenetic signatures in association with birth weight discordance.

**Results:**

Our association analysis revealed no CpG site with genome-wide statistical significance (FDR < 0.05) for either qualitative (larger or smaller) or quantitative discordance in birth weight. Even with selected samples of extremely birth weight discordant twin pairs, no significant site was found except for 3 CpGs that displayed age-dependent intra-pair differential methylation with FDRs 0.014 (cg26856578, p = 3.42e-08), 0.0256 (cg15122603, p = 1.25e-07) and 0.0258 (cg16636641, p = 2.05e-07). Among the three sites, intra-pair differential methylation increased with age for cg26856578 but decreased with age for cg15122603 and cg16636641. There was no genome-wide statistical significance for sex-dependent effects on intra-pair differential methylation in either the whole samples or the extremely discordant twins.

**Conclusions:**

Genome-wide DNA methylation profiling did not reveal epigenetic signatures of birth weight discordance although some sites displayed age-dependent intra-pair differential methylation in the extremely discordant twin pairs.

**Electronic supplementary material:**

The online version of this article (doi:10.1186/1471-2164-15-1062) contains supplementary material, which is available to authorized users.

## Background

According to the Barker hypothesis of Developmental Origins of Health and Disease (DOHaD), susceptibility to certain common diseases such as hypertension and type 2 diabetes may be related to poor environmental condition early in life [1, 2]. Poor fetal and/or early postnatal condition during critical growth phases may alter the structural and physiologic functional development of vital organs regulating blood sugar and blood pressure, thus predetermining the susceptibility to hypertension and diabetes in adulthood. Poor fetal nutrition may lead to a “thrifty” phenotype that selectively protects the growth of the most vital organs (e.g., brain) at the expense of the less vital ones (e.g., pancreatic β-cell mass, nephron cell mass) during fetal development [2]. Such fetal adaptations increase the chance of survival in an adverse intrauterine environment, but they impair metabolic functional capacity in coping with metabolic stressors in postnatal life. Animal studies have provided strong evidence that poor fetal nutrition leads to low birth weight in offspring and increased blood pressure in adulthood [3, 4]. Due to multiple practical reasons, direct evidence connecting prenatal environmental condition and adult life health consequences in humans has been sparse.

In the literature, controversial results have been reported for linking early life events with disease at adult ages. In a recent epidemiological study on the birth weight discordant monozygotic twins included in the present study, Frost *et al*. [5] found no effect of low birth weight on glucose metabolism. Likewise, Petersen *et al.* [6] reported no indication of increased occurrence of type 2 diabetes among twins who on average are about 1 kg lighter at birth than singletons. Despite the negative findings, significant associations have been frequently reported. For example, Iliadou *et al.* [7] reported an inverse association between birth weight and the risk of type 2 diabetes; Wannamethee *et al*. [8] found lower birth weight in monozygotic twins with type 2 diabetes compared to their co-twins. The inconsistent findings from epidemiological studies on disease endpoints call for new approaches in the search for evidences of an association between intrauterine growth and metabolic dysfunction.

Epigenetics is the study of changes in gene expression or cellular phenotype, caused by mechanisms other than changes in the underlying DNA sequence. As opposed to traditional epidemiologic approaches that seek indirect evidence by associating environmental exposure with disease outcome, epigenetic epidemiology searches for direct molecular evidence that links environmental factors to epigenetic regulation of genes and to the development of diseases [9]. The molecular mechanism of epigenetic control over gene activity involves DNA methylation, chromatin modification, regulatory non-coding RNAs (ncRNAs) including post-transcriptional regulation by microRNA (miRNA), etc. Of the various epigenetic regulation mechanisms, DNA methylation is the major form of epigenetic modification that is most robust and readily measurable using high throughput techniques, for example the Illumina’s Infinium HumanMethylation450 Beadchip assay, for methylation profiling at genome scale [10].

Monozygotic (MZ) twins share genetics and uterus, albeit not necessarily the same intrauterine environment [11]. Therefore, MZ twins and especially birth weight discordant MZ pairs provide an extraordinary opportunity to investigate the association between early life factors and adult life health while controlling for not only genetics but also postnatal environments [9]. In a recent study, Souren *et al.* [12] conducted genome-wide DNA methylation profiling in saliva on 17 pairs of MZ twins discordant for birth weight and reported indistinguishable patterns of DNA methylation. Considering the limited power in their study due to very small sample size, we performed a relatively large-scale epigenomic association analysis on blood samples from adult Danish MZ twins discordant for birth weight with the aim of identifying genomic regions under differential methylation in association with birth weight discordance.

## Results

### Samples of birth weight discordant twins

**Figure 1:**
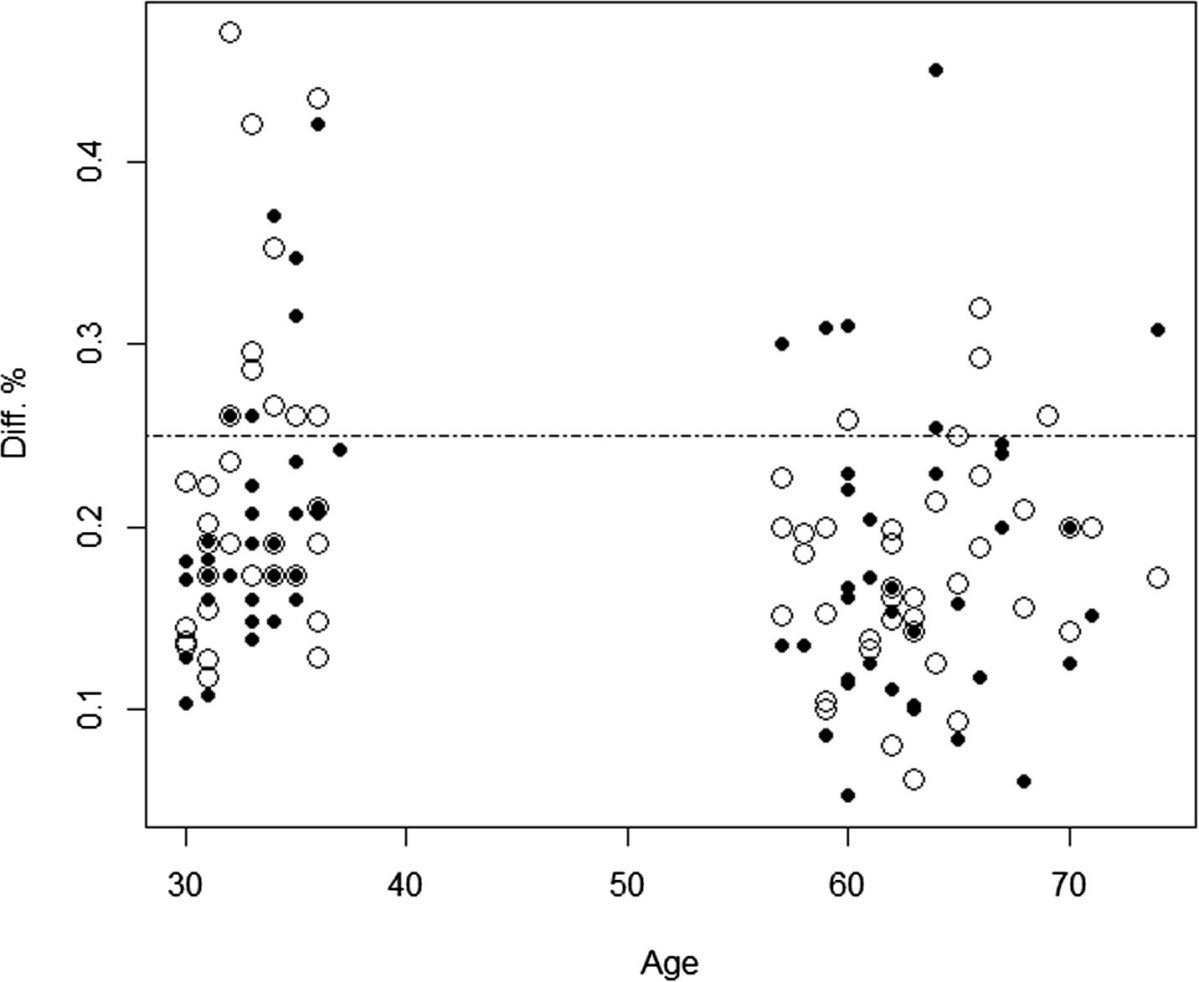
**Birth weight discordant twin pairs displayed by plotting ∆bw% against age with male twin pairs represented by solid and female twin pairs by empty circles.** The dash-dotted line shows the cut-off of ∆bw% = 25%.

In Table 1, we show the descriptive statistics of the 150 pairs of MZ twins. Among them, 78 pairs were male and 72 pairs female twins. At recruitment, the youngest twin pair was 30 years and the oldest 74 years. From the age distribution, as shown in Figure 1, there are two age groups of twins with age intervals from 30 to 37 in the younger twins and from 57 to 74 in the older twins. The age gap of 20 years was caused by a “gap” in the registered data available for birth weight. Intra-pair birth weight difference ranges from 200 to 1250 grams with a median of 500 grams. Based on absolute birth weight difference, we calculated percentage of difference in birth weight (∆bw%) as absolute birth weight difference divided by the birth weight of the larger twin, i.e.

**Table 1:** **Descriptive statistics of the twin samples**

	All twins	Young twins*	Old twins*
Age, years			
Min	30	30	57
Max	74	36	74
Median	57	33	63
Sex, pairs			
Male	78	40	38
Female	72	33	39
Birth weight, grams			
Min	1100	1125	1100
Max	4625	4625	4150
Mean	2528	2587	2577
Birth weight discordance, %			
Min	5.26	10.34	5.26
Max	47.06	47.06	45.00
Mean	17.73	19.05	17.51
Gestational age, weeks			
Min		33	
Max		42	
Median		39	

*Young twins were born after 1973 and old twins born before 1973. Gestational age was available after 1973 from The Danish Medical Birth Registry.

As shown by Figure 1 and Table 1, the calculated ∆bw% ranges from 5.26% to 47.06% with a median of 17.7%. From the figure, we see that the male (small solid circles) and female (empty circles) twins are evenly distributed over age and by ∆bw%. However, the degree of discordance seems higher in the young twins than in the old twins. This is confirmed by statistical testing with a p value of 0.0044 (*t* = 2.89, *df* = 145).

### EWAS on birth weight discordant twins

**Figure 2:**
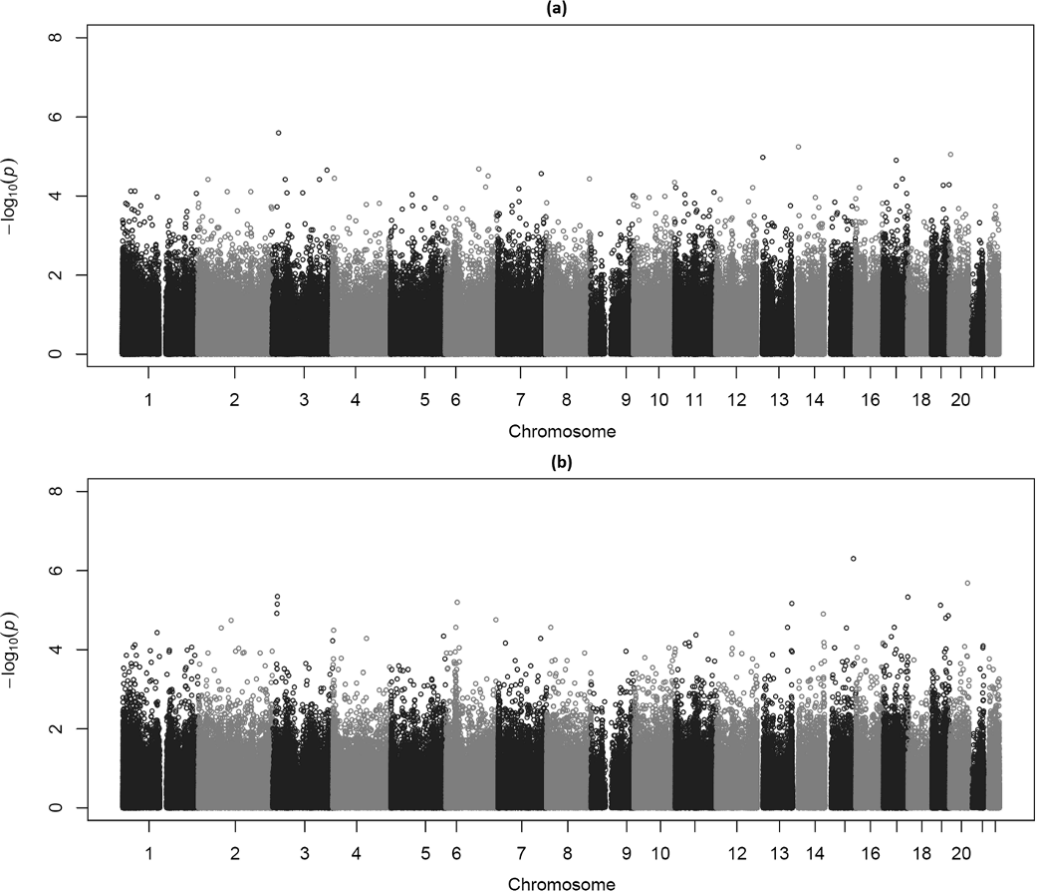
**Manhattan plots showing p values of single CpGs across the genome obtained by EWAS on qualitative (a) and quantitative (b) discordance in 150 pairs of twins.**

DNA methylation data was analysed by a linear mixed effects model [13] that contained fixed effects for qualitative (larger or smaller) as well as quantitative or degree of birth weight discordance. The model was capable of estimating the effect on DNA methylation from birth weight discordance in general defined as a binary trait and from degree of birth weight discordance defined as a quantitative trait (see Statistical analysis section). In addition to the fixed effect covariates described in the method section, our mixed model specified sample plate, well, and sentrix position as random variables to account for batch effect and random effects due to sample allocation on the Infinium BeadChip in the array experiment. Results from our EWAS revealed no CpG sites significantly associated with either qualitative or quantitative (∆bw%) birth weight discordance at genome-wide level pre-defined as false discovery rate (FDR) < 0.05. Although no genome-wide statistical significance was seen, the CpG site cg20716652 located in the gene body of the *RGMA* gene on chromosomes 15 had the lowest p value of 5.06e-07 (β = -1.70) corresponding to a FDR of 0.21 for degree of birth weight discordance. DNA methylation level at this site seemed to become higher in the smaller twins than in the bigger twins with increasing percentage of discordance. Figure 2a is the Manhattan plot for EWAS on qualitative discordance and Figure 2b for quantitative discordance.

### EWAS on extreme birth weight discordant twins

**Figure 3:**
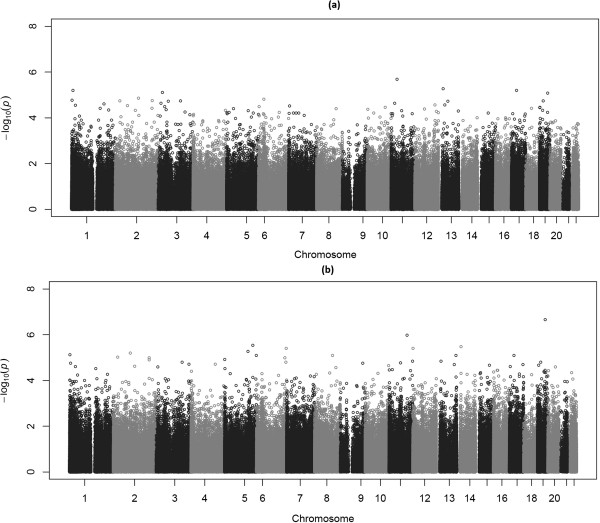
**Manhattan plots showing p values of single CpGs across the genome obtained by EWAS on qualitative (a) and quantitative (b) discordance in 28 pairs of twins.**

As shown in Table 1, there is a big difference in the degree of birth weight discordance among the twin pairs ranging from 5 to 47%. With this in mind, we performed our second EWAS on samples with extreme birth weight discordance. To do that we selected twin pairs with ∆bw% >25% since a birth weight difference of over 25% is supposed to represent a pathologic process [14, 15]. A total of 28 pairs were selected for further analysis with a median of 30% in birth weight difference. The same mixed model was applied for analysis of the extremely discordant twins. Figure 3 displays the Manhattan plots for the p values from EWAS on qualitative (Figure 3a) and quantitative (Figure 3b) discordance. Again, no site showed genome-wide statistical significance for qualitative or quantitative discordance. The CpG site (cg05275595) had the lowest p value of 2.18e-07 (β = -0.84, FDR 0.09). The site is located in the first exon of the *CD177* gene on chromosome 19. The smaller twins were characterized by higher methylation at this site than the bigger twins with increasing percentage of birth weight discordance.

### Age- and sex-dependent intra-pair differential DNA methylation

In Table 1, the median ages for the young and the old twins have a gap of 30 years. By specifying age as a pair-specific fixed effect covariate, we were able to examine if there were significant intra-pair differential DNA methylation patterns that were age-dependent. Likewise, sex-dependent intra-pair differential methylation can also be captured by our model with sex included as a pair-specific fixed effect covariate. Our EWAS on the whole sample did not find any age- or sex-dependent pattern on intra-pair differential methylation at genome-wide level (Additional file 1: Figure S1). However, for the selected samples of extremely discordant twins, 3 CpG sites showed genome-wide statistical significance for age-dependent intra-pair differential methylation with FDR 0.014 (cg26856578, β = 0.003, p = 3.42e-08), 0.0256 (cg15122603, β = -0.002, p = 1.25e-07) and 0.0258 (cg16636641, β = -0.018, p = 2.05e-07) (Additional file 2: Figure S2). Among the 3 sites, cg26856578 became highly methylated with increasing age in the bigger than in the smaller twins, but the pattern was reversed for cg15122603 and cg16636641, i.e., higher methylation in the smaller than in the bigger twins. The 3 CpGs are located on chromosomes 13 (cg26856578), 5 (cg15122603) and 18 (cg16636641 in the promoter region of a protein coding gene *ZCCHC2*). The 2 sites, cg26856578 and cg15122603 were not linked to any genes. The lowest p value of 2.73e-07 for sex-dependent effects was achieved by cg03813964 on chromosome 2 with FDR = 0.11 (β = -0.052). There was no site exhibiting genome-wide statistical significance for sex-dependent effect in the selected samples (Additional file 2: Figure S2).

## Discussion

We have conducted a genome-wide DNA methylation profiling in whole blood samples of identical twins discordant for birth weight. Although with a large sample size of 150 twin pairs, our EWAS did not detect any CpG sites differentially methylated at genome-wide statistical significance, neither for qualitative nor for quantitative concordance. Even for the extremely discordant twins, no genome-wide statistical significance was found for qualitative or quantitative discordance. Overall, our results seem to support the conclusion drawn by Souren *et al.* [12], i.e. there is no obvious epigenetic signature for birth weight discordance in the adult twins. In a recent study, Engel *et al*. [16] reported 19 significant CpG sites associated with birth-weight in the cord blood of a large sample of 1046 infants. The lack of replication from our study could be due to the fact that the two studies focused on different stages of life and the measured DNA methylation status in infants does not necessarily represent that in adults. Similar studies in the adult population should provide valuable validation to our conclusion.

Contrary to the analytical method used by Souren *et al*. [12], our mixed effects model is capable of measuring age- and sex-dependent effects on intra-pair differential methylation. Although no age- or sex-dependent pattern was found in the whole sample of 150 twin pairs, in the extremely discordant twin pairs with ∆bw% >25%, 3 CpG sites had genome-wide statistical significance for their age-dependent effects on intra-pair differential DNA methylation with FDRs 0.014 (cg26856578, p = 3.42e-08), 0.0256 (cg15122603, p = 1.25e-07) and 0.0258 (cg16636641, p = 2.05e-07). The odds ratio for intra-pair differential methylation was calculated as 1.04, 1.19 and 0.84, respectively, for a period of 10 years. Although with a small effect size, the significant age-dependent effect indicates that the epigenetic impact of birth weight discordance could be expressed progressively as either increasing or decreasing DNA methylation levels with increasing age.

Using the same study population of twins as the one included in the present study, Frost *et al*. [5] performed an epidemiological analysis of birth weight discordance and glucose metabolism and found no evidence for a detrimental effect of low birth weight on glucose metabolism including beta cell function, insulin resistance, insulin sensitivity and acute insulin response, even in twin pairs with a large intra-pair difference in birth weight. In general, our results are in agreement with these epidemiological findings, although 3 CpGs showed genome-wide statistical significance for their age-dependent effect in the extremely discordant twins. The significant age-dependent effect on intra-pair differential methylation from the extremely discordant twins prompted us to speculate whether they were distinct from the rest of twin pairs. So, we examined whether the extremely discordant twins were different from the rest of twins in term of preterm birth defined as birth with gestational age shorter than 37 complete weeks. In Additional file 3: Table S1, we show the count of twin pairs stratified by gestational age (>37 weeks or ≤37 weeks) and by ∆bw% (≥25 or <25) in young twins, a group whose gestational ages were recorded by the Danish Medical Birth Registry starting from early 1970s. The statistical test showed that the proportion of preterm twin pairs in the extremely discordant twin pairs (10 out of 17 or 58.8%) is significantly higher than that in the rest of the twins (15 out of 56 or 26.8%) (χ2 = 5.944, p = 0.015). In the older twins, exact gestational age was not available, and only for some of the twins, estimated gestational age was known. Of the 10 old twin pairs with ∆bw% ≥25, five had missing data for gestational age and the remaining 5 all belonged to the group with a gestational age ≤37 estimated weeks. Altogether, the extremely discordant twins were significantly over-represented by preterm twins who might have different clinical characteristics as compared to other birth weight discordant twins.

The degree of methylation could differ between monochorionic and dichorionic monozygotic twins, presumably because variation in nutrition is lower in the latter [17]. Information on chorionicity was not available in our study, and we cannot exclude the possibility that the results would have been different, had we investigated either mono- or dichorionic monozygotic twins. In addition, monozygotic twins may not be entirely genetically identical. Mosaicism for de novo mutations [18], differences in copy-number variations [19], and post-zygotic mutations [13] may all contribute to genetic differences between monozygotic twins and, potentially, the pattern of DNA methylation under investigation.

In the present study, DNA methylation was assessed in white blood cells, i.e. cells derived from the mesoderm, whereas, Souren *et al.* [12] used saliva, which comprises cells originating from both the mesoderm and the ectoderm (white blood cells and epithelium, respectively). The relative importance of the tissue used for assessment of the methylation profile is uncertain. The heritability of the methylome is lower in white blood cells than in buccal tissue [17], and the epigenome differed significantly between the four tissues investigated in monozygotic twins by Ollikainen *et al.* [13], suggesting that selection of tissue may influence the investigations. We have no knowledge of any potential difference in DNA methylation that could be caused by intrauterine growth impairment in relevant tissues such as muscle, fat and liver, and emphasize that the finding from the current study only reflects changes observed in whole blood samples and may not be generalized to other tissues. Since the difference in birth-weight was observed at end of gestation, the epigenetic patterns associated with it could then be tissue-specific and consequently too late to be seen in the blood. In this regard, biologically more relevant tissues could help with identifying the epigenetic patterns associated with birth-weight discordance.

Overall, our results did not show significant epigenetic profiles associated with birth weight discordance in identical twins, a conclusion that is in agreement with Frost *et al*. [5] who reported no detrimental effect of low birth weight on glucose metabolism in the same twin samples as those used in the current study. Meanwhile, our study is limited by the use of whole blood as target tissue, limited sample size for genome-wide association analysis and low coverage of the methylation chips. Future studies which include larger study populations and samples from biologically appropriate tissues using e.g. next generation sequencing techniques should be able to provide more evidence of whether birth weight has any effect on later life DNA methylation and to verify the epigenetic basis of DOHaD.

## Conclusions

Results from an EWAS based on our relatively large sample size of twins did not reveal significant epigenetic signature for birth weight discordance. Even in the extremely birth weight discordant twin pairs who were dominated by preterm birth, no statistically significant results were found except for some age-dependent effects. In conclusion, our data did not show molecular evidence of differential DNA methylation in adult twin pairs discordant for birth weight.

## Methods

### Experiment design and sampling of twins

This study was conducted using the discordant MZ twin design in which identical twin pairs discordant for birth weight were sampled for epigenetic profiling at adult ages. The design provides a perfect match for the genetic make-ups and rearing environments within each pair of MZ twins. The design minimizes the effects from both known (genetics, age, sex) and unknown potential confounding factors [20]. Our twins were sampled based on information from the Danish Twin Registry and records on birth weight retrieved from the Danish Birth Record Registry for the young twins and from midwife records for the old twins [5]. A total of 153 pairs of the most discordant MZ twin pairs were identified and included in the study. Zygosity information based on physical resemblance from the Danish Twin Registry was further confirmed using 12 highly polymorphic microsatellite markers. Informed consent to participate in the study was obtained from all participants. The study was approved by The Regional Scientific Ethical Committees for Southern Denmark (S-20090033) and conducted in accordance with the Helsinki II declaration.

### Blood sample collection and genomic DNA extraction

EDTA-anticoagulated blood samples were collected between 07:00 and 09:00 hours after an overnight fast of at least 8 hours. The samples were processed immediately or kept at room temperature for up to 24 hours, until further processing. The blood was centrifuged at 1000 g for 10 min, and buffy-coat was frozen in aliquots at -80°C. DNA was isolated from the buffy-coats using the salt precipitation method applying either a manual protocol or a semi-automated protocol based on the Autopure System (Qiagen, Hilden, Germany). Bisulphite treatment of 500 ng template genomic DNA was carried out with the EZ-96 DNA methylation kit (Zymo Research, Orange County, USA) following the manufacturer’s protocol.

### Genome-wide DNA methylation analysis

Whole genome DNA methylation level was measured using the Illumina’s Infinium HumanMethylation450 Beadchip assay (Illumina, San Diego, CA, USA) at the Leiden University Medical Center. The array allows simultaneous measurement of DNA methylation status at more than 480,000 CpG sites across and beyond gene and CpG island regions in the human genome. The laboratory work for the assay was performed according to the manufacturer’s instructions. In order to minimize the batch effect on intra-pair DNA methylation differences, twins of each pair were processed together on same array (each array contained 12 samples, i.e. 6 pairs of twins). Data normalization was done using the free R package *minfi* which employs subset quantile within-array normalization [21]. At each CpG site, DNA methylation level was summarized by calculating a “beta” value defined by the Illumina’s formula as β = M/(M + U + 100).

### Estimating and adjusting cell composition

When whole blood DNA is used in epigenetic association analysis, cell composition can serve as a confounder as it can change as a result of aging or disease status. To deal with the problem, we estimated cell composition in each individual for 6 blood cell types: CD8T, CD4T, natural killer cell, B cell, monocyte, and granulocyte using free R package *minfi*. The package estimates cell composition based on the DNA methylation data measured on a whole blood sample and published cell-type-specific DNA methylation data using an approach proposed by Houseman *et al*. [22]. Based on the estimates, the effect of cell composition was first removed by fitting a regression model that regresses measured DNA methylation level at a CpG site on the estimated cell composition and the residuals were kept for next step association analysis.

### Quality control (QC) at probe level

The probe level QC was realized by taking advantage of technical replicates performed on one pair of twins with 4 replicates for each singleton (i.e. genome-wide DNA methylation repeatedly measured on 4 DNA samples from each twin). With the 8 technical replicates (4 on each twin in the pair), we first fitted a mixed model adjusting for the mean DNA methylation level in each twin (fixed effect) with technical replicates from each twin treated as one cluster (random effect). We then calculated standard errors for the residuals (random error) from the 8 replicates for each probe tested on the array. The calculated standard error reflects the variation of the random errors from 8 technical replicates, in other words, the variability of each probe. This was done for data on the 8 replicates pre-processed by different methods including β values provided by *minfi* using the Illumina’s formula without normalization, subset-quantile within array normalization (SWAN), quantile normalization upon SWAN (QNN). As shown in Additional file 4: Figure S3a, the SWAN and QNN methods gave the lowest error and performed equally well. Our subsequent analysis was therefore performed on data normalized by SWAN. Based on the distribution of the standard errors for all probes on the array (Additional file 4: Figure S3a), we defined probes with standard error > 0.05 as unstable probes.

Furthermore, we also control probe quality using the detection p value defined as the proportion of samples reporting background signal levels for both methylated and unmethylated channels. The detection p was calculated by *minfi*. A β value with its assigned detection p value > 0.01 was treated as missing. CpG sites with more than 5% missing data were dropped from the subsequent analysis. Of the 485577 CpGs on the array, 484895 were kept after QC at probe level with only 682 CpGs failed.

### QC at sample level

We made use of twin correlation on genome-wide DNA methylation pattern as a mean of quality control at sample level. The idea is that, since high intra-pair correlation on genome-wide DNA methylation is expected for identical twins, who presumably share the same genetic predisposition, exceptional dissimilarity within a pair is an indication of poor sample quality due to, for example, the fact that one of them suffers from a disease or has some other extreme phenotype (e.g. a very different blood cell distribution). In Additional file 4: Figure S3b, we show the calculated intra-pair correlation on DNA methylation for each pair plotted against the age of each pair. To obtain the distribution for a random correlation, we randomly formed pseudo-twin pairs and calculated the correlation for 10000 replicates. The dashed and dash-dotted blue lines represent the 95% intervals of the random distributions for the old (0.973-0.993) and young (0.978-0.993) twins, respectively. The figure shows that there are 3 pairs of twins displaying intra-pair correlation exceptionally lower than that from pseudo-twin pairs suggesting poor sample quality in one or both twins in the 3 pairs. The 3 pairs were removed from subsequent analyses leaving our sample size with 150 pairs.

In addition to twin correlation on whole genome DNA methylation, Additional file 4: Figure S3b also displays a slight trend of decrease in the correlation with increasing age, a pattern that has been reported by Talens *et al.* [23] in monozygotic twin pairs. For that reason, the random distribution of twin correlation was generated for the young and the old groups separately. Most importantly, the age pattern in the twin correlation also suggests the importance of adjusting for age in assessing the association between DNA methylation status and birth weight discordance.

### Statistical analysis

In order to analyse epigenetic data from birth weight discordant twins, we introduced a mixed effects model [20] with fixed effects of age, sex, birth weight discordance in percentage defined above, random effects for batches (plate and well) and sample location on the array.


Here Me(+) and Me(-) are DNA methylation levels measured in the bigger and the smaller twins of in a pair; βs are the slopes for fixed effect variables (*x*_*1*_ to *x*_*n*_), *y*_*1*_ to *y*_*m*_ are the random effect variables including batch effects. In this model, the intercept α is an important parameter because it stands for the mean fold change in DNA methylation level between the smaller and the bigger twins for the effect of qualitative discordance. The intercept α represents the mean level of intra-pair methylation difference when all fixed effect variables are set to zero. The βs are regression coefficients for pair-specific variables, e.g. age, sex, etc. Note that, although the pair-specific variables are matched out in the discordant twins design, their effects on intra-pair difference in DNA methylation status cannot be ignored. One very good example is the age pattern of twin correlation on genome-wide DNA methylation as shown in Additional file 4: Figure S3b. Most importantly, the model includes ∆bw% as a pair-specific variable assuming that the degree of birth weight discordance, i.e. quantitative discordance, affects the extent of intra-pair difference in DNA methylation level. This means that our model includes parameters standing for the effects of both qualitative and quantitative discordance while adjusting for important covariates (age, sex) and for random effects. With the estimated regression coefficients, the fold change or odds ratio for intra-pair differential methylation for one unit change in a corresponding covariate can be calculated by taking the exponential of the regression coefficient.

All statistical analyses were performed using the free R statistical software (http://www.r-project.org) and the fitting of mixed effect model was realized by applying the R package *lmerTest* (http://cran.r-project.org/web/packages/lmerTest) which provides a p value calculation for statistical significance of the fixed effect covariates. Correction for multiple testing was done by estimating the false discovery rate (FDR) [24] using the *p.adjust()* function in R. Genome-wide statistical significance was defined as FDR < 0.05.

### Availability of supporting data

Both raw and processed DNA methylation data have been deposited to the NCBI GEO database http://www.ncbi.nlm.nih.gov/geo/ under accession number GSE61496.

## Electronic supplementary material

Additional file 1: Figure S1: Manhattan plots showing p values for age (a) and sex (b) dependent effects of single CpGs across the genome obtained by EWAS on birth weight discordance in 150 pairs of twins. (BMP 8 MB)

Additional file 2: Figure S2: Manhattan plots showing p values for age (a) and sex (b) dependent effects of single CpGs across the genome obtained by EWAS on birth weight discordance in 28 pairs of extremely discordant twins. (BMP 8 MB)

Additional file 3: Table S1: Younger age twin pairs stratified by gestational age and degree of birth-weight discordance. (DOCX 14 KB)

Additional file 4: Figure S3: Quality control at probe (left panel) and sample (right panel) levels. The curves with different colours show errors calculated for data on the 8 replicates pre-processed by different methods including β values provided by minfi without normalization, SWAN, quantile normalization upon SWAN (QNN). As shown in left panel, the SWAN and QNN methods gave the lowest error and performed equally well. The density plot of probe-specific standard error showed very high concentration of probes with low errors and very small number of probes with standard error > 0.05. In the right panel, three pairs of twins showed intra-pair correlation on genome-wide DNA methylation lower than random and were dropped from subsequent analyses. (BMP 4 MB)
