# Supplementary figures and images for: Epigenetic signature of birth weight discordance in adult twins
Source: BMC Genomics. 2014 Dec 4;15(1):1062. doi: 10.1186/1471-2164-15-1062 (PMC4302120; doi:10.1186/1471-2164-15-1062)

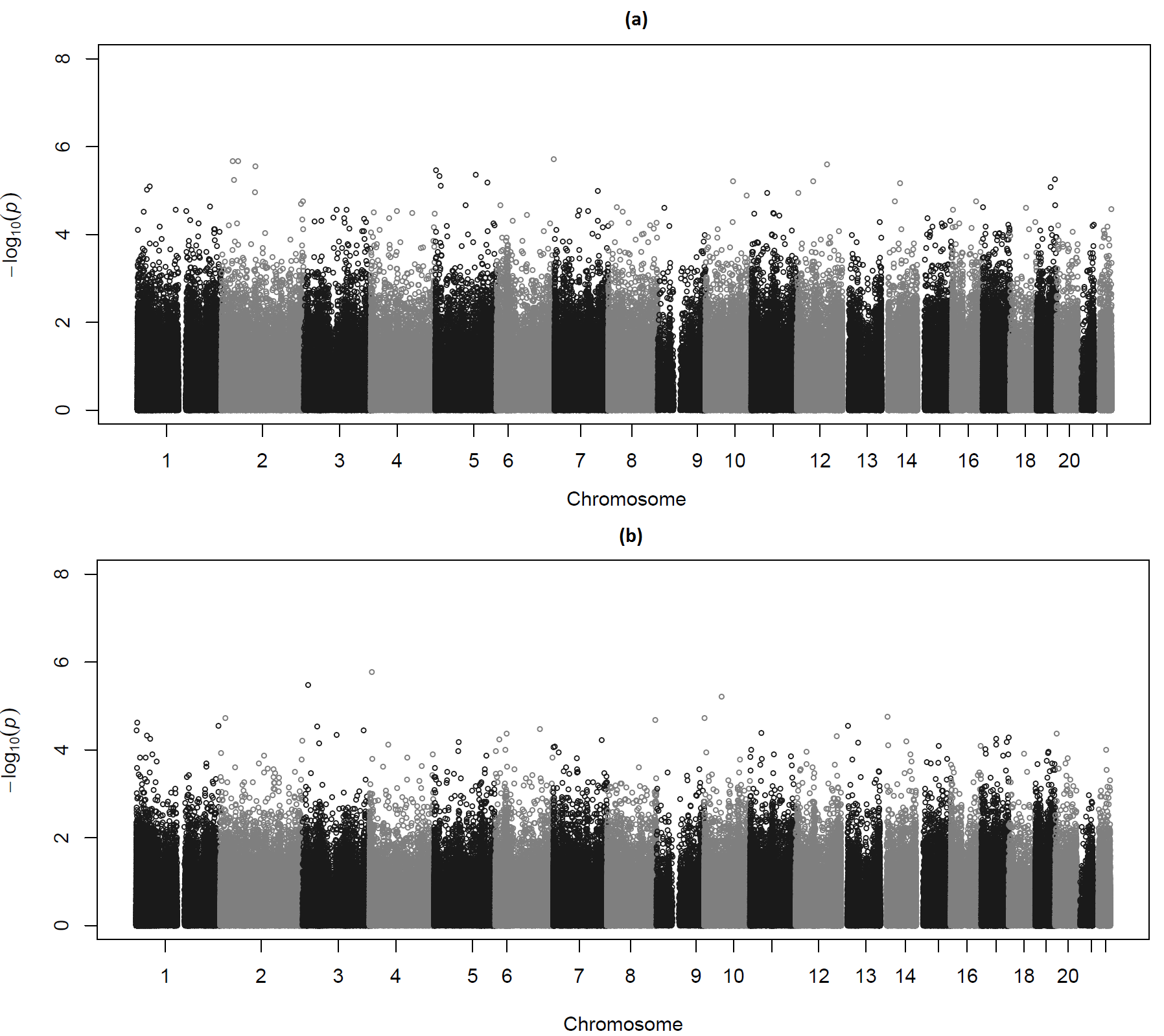

Supplement: Supplementary file 1 — Additional file 1: Figure S1: Manhattan plots showing p values for age (a) and sex (b) dependent effects of single CpGs across the genome obtained by EWAS on birth weight discordance in 150 pairs of twins. (BMP 8 MB) [file 12864_2014_6769_MOESM1_ESM.bmp]

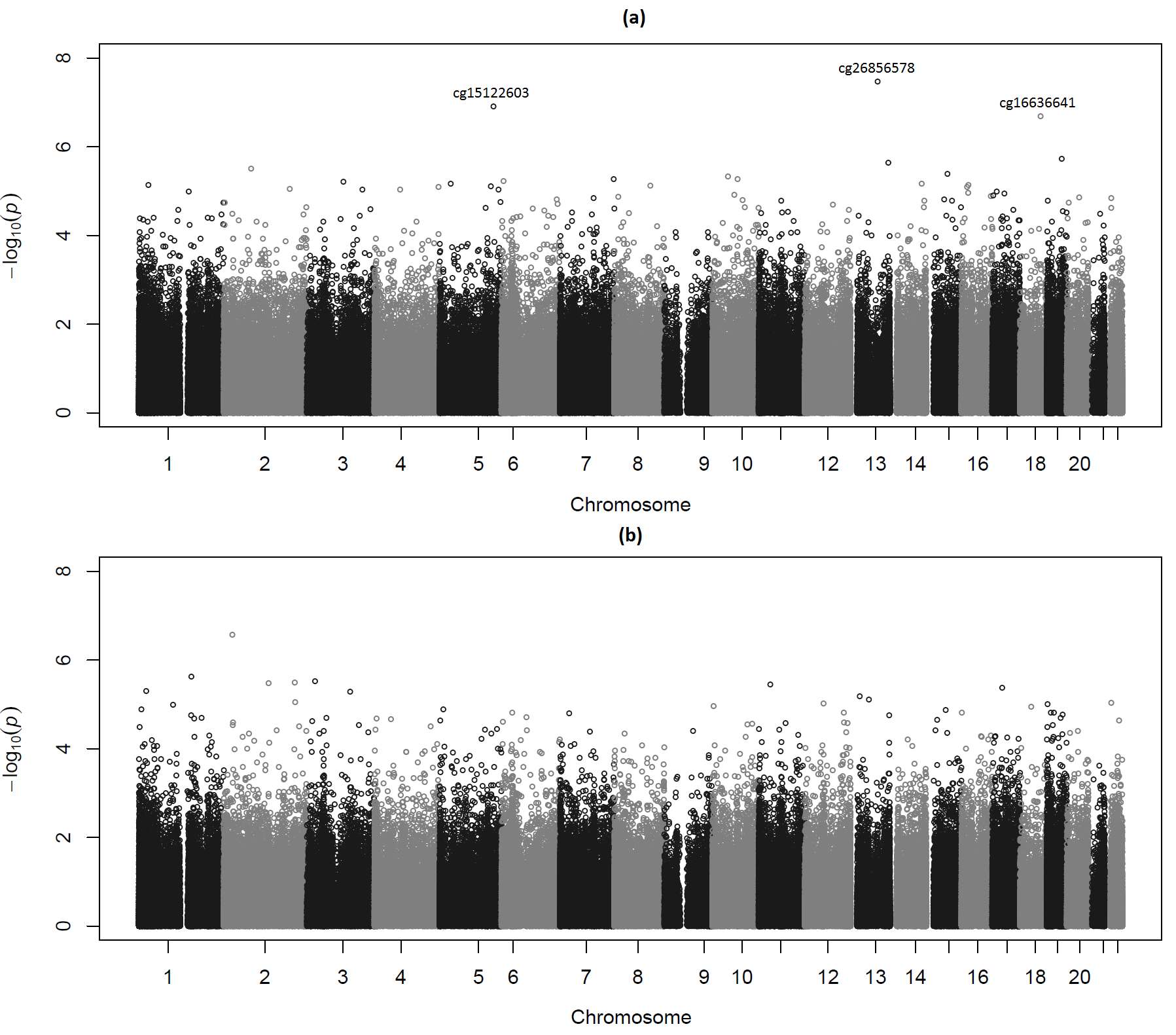

Supplement: Supplementary file 2 — Additional file 2: Figure S2: Manhattan plots showing p values for age (a) and sex (b) dependent effects of single CpGs across the genome obtained by EWAS on birth weight discordance in 28 pairs of extremely discordant twins. (BMP 8 MB) [file 12864_2014_6769_MOESM2_ESM.bmp]

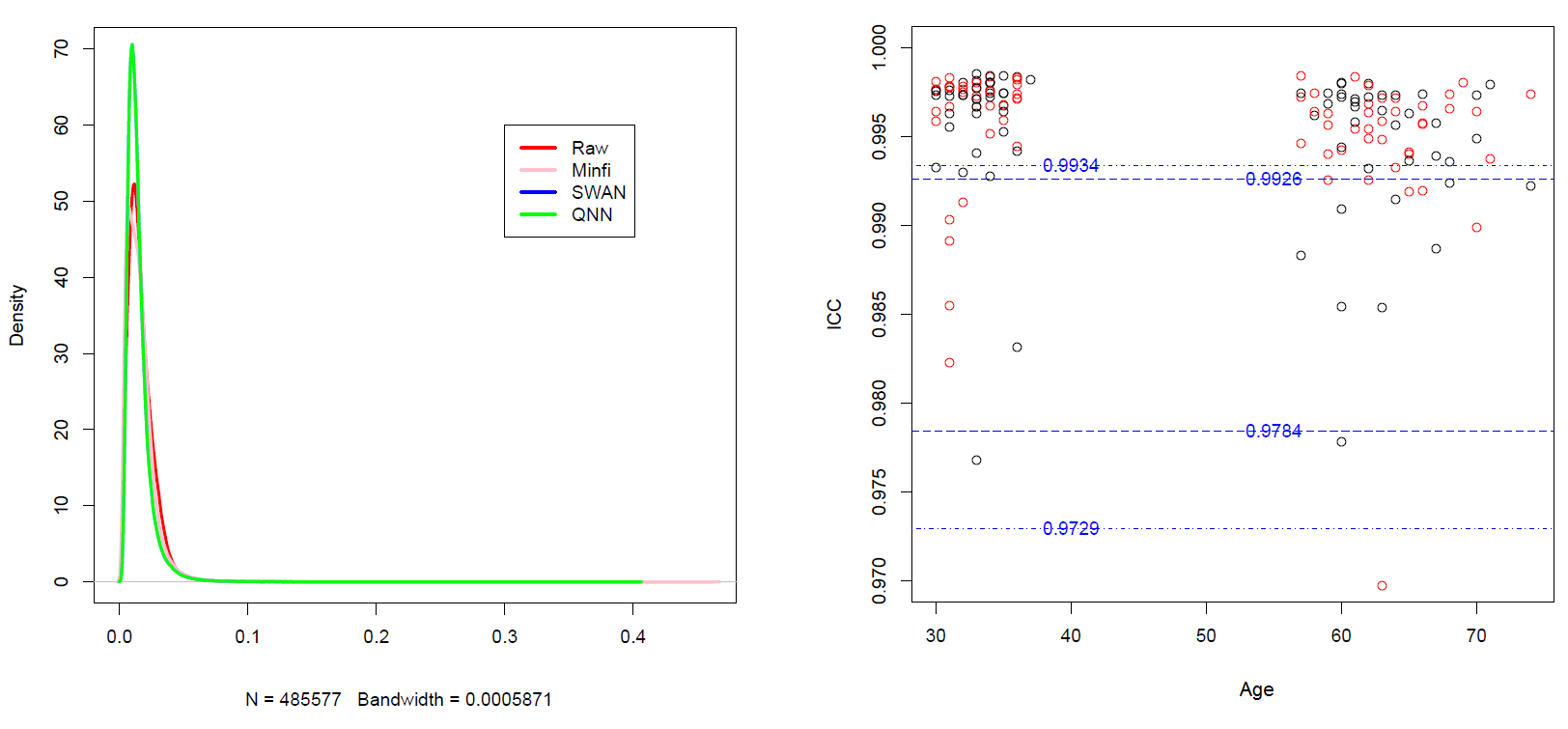

Supplement: Supplementary file 4 — Additional file 4: Figure S3: Quality control at probe (left panel) and sample (right panel) levels. The curves with different colours show errors calculated for data on the 8 replicates pre-processed by different methods including β values provided by minfi without normalization, SWAN, quantile normalization upon SWAN (QNN). As shown in left panel, the SWAN and QNN methods gave the lowest error and performed equally well. The density plot of probe-specific standard error showed very high concentration of probes with low errors and very small number of probes with standard error > 0.05. In the right panel, three pairs of twins showed intra-pair correlation on genome-wide DNA methylation lower than random and were dropped from subsequent analyses. (BMP 4 MB) [file 12864_2014_6769_MOESM4_ESM.bmp]
