# Supplementary material for: Epigenetic signature of birth weight discordance in adult twins
Source: BMC Genomics. 2014 Dec 4;15(1):1062. doi: 10.1186/1471-2164-15-1062 (PMC4302120; doi:10.1186/1471-2164-15-1062)
Supplement: Supplementary file 3 — Additional file 3: Table S1: Younger age twin pairs stratified by gestational age and degree of birth-weight discordance. (DOCX 14 KB) [file 12864_2014_6769_MOESM3_ESM.docx]

Table S1 Younger age twin pairs stratified by gestational age and degree of birth-weight discordance

| ∆bw% | Gestational age | |  |
| --- | --- | --- | --- |
|  | >37 | ≤37 |  |
| ≥25 | 7 | 10 | 17 |
| <25 | 41 | 15 | 56 |
| Total | 48 | 25 | 73 |
